# Effect of STOX1 on recurrent spontaneous abortion by regulating trophoblast cell proliferation and migration via the PI3K/AKT signaling pathway

**DOI:** 10.1002/jcb.28112

**Published:** 2018-12-12

**Authors:** Zhifang Li, Guiju Zhou, Longfan Jiang, Huifen Xiang, Yunxia Cao

**Affiliations:** ^1^ Reproductive Medicine Center, The First Affiliated Hospital, Anhui Medical University Hefei China; ^2^ Anqing Municipal Hospital, Anhui Medical University Anqing China; ^3^ Department Gynecology, The Second Affiliated Hospital, Anhui Medical University Hefei China

**Keywords:** migration, PI3K/Akt signaling pathway, proliferation, recurrent spontaneous abortion (RSA), storkhead box 1 (STOX1), trophoblast

## Abstract

STOX1 is a transcription factor that is implicated in the high prevalence of human gestational diseases. It has been studied in various types of gestational diseases using different molecular and cellular biological technologies. However, the effect and detailed mechanism of storkhead box 1 (STOX1) in recurrent spontaneous abortion (RSA) remain unknown. This study aimed to explore the effect and detailed mechanism of STOX1 in human trophoblast cells. The result showed that downregulation of STOX1 by short hairpin RNA (shRNA) led to a decrease in proliferation and migration in HTR‐8/SVneo cells, while it induced the apoptosis of HTR‐8/SVneo cells. Moreover, the result showed that trophoblast cells expressed lower levels of pAKT and p85 subunits after treatment with STOX1 shRNA when compared with control. However, overexpression of STOX1 obviously increased the pAKT and p85 protein expressions. Transfection of pcDNA‐AKT plasmid increased cell proliferation and migration in HTR‐8/SVneo cells while suppressed the apoptosis of HTR‐8/SVneo cells. Furthermore, inhibition of the PI3K/Akt pathway by a specific inhibitor promoted cell apoptosis and aggravatedly suppressed cell proliferation and migration of HTR‐8/SVneo cells. On the other hand, upregulation of the PI3K/Akt pathway could increase the relative expression level of Bcl‐2 and decrease the relative expression levels of Bax and Bim, while inhibition of the PI3K/Akt pathway led to adverse results. Our results demonstrated that inhibition of STOX1 could suppress trophoblast cell proliferation and migration, while promote apoptosis through inhibiting the PI3K/Akt signaling pathway. These findings might provide a new fundamental mechanism for regulating RSA and could be used to prevent and treat RSA in clinic.

## 1 INTRODUCTION

Recurrent spontaneous abortion (RSA), an important factor affecting human reproduction, leads to various complications and even infertility, which seriously threatens women's health around the world.^1^ The American Society of Reproductive Medicine defines two or more failed clinical pregnancies as RSA. While the Royal College of Obstetricians and Gynecologists and the European Society of Human Reproduction and Embryology stipulate that RSA should be defined as more than three consecutive spontaneous abortions before 20 weeks of pregnancy. According to statistics, 15%‐25% of pregnant women miscarry, of which at least 5% of pregnant women experience two abortions, and 1% of pregnant women even experience more than three miscarriages.^2^, ^3^, ^4^ A number of studies have shown that factors including cytogenetic abnormalities, anatomic irregularities, endocrine disorders, infection, autoimmunity, atypical blood clotting, sperm quality, and environmental factors can possibly induce miscarriage.^5^, ^6^, ^7^, ^8^ Recently, the dysfunction and dysregulated expression of correlative genes were reported to play a pivotal role in the risk of miscarriage.^9^, ^10^ A previous study demonstrated that unexplained RSA was probably associated with Foxp3 dysfunction and its abnormal expression, which could suppress the regulatory function of Treg cells and resulted in the failure of fetal‐maternal immunologic tolerance.^11^ In addition, the aberrant expression of ARNT‐like protein 1 could regulate RSA through inhibiting trophoblast migration and invasion by the SP1‐DNMT1/DAB2IP pathway.^12^ Although the related genes and its polymorphism were found to be associated with the RSA, the definite causes and detailed mechanism of RSA remain unknown.

Storkhead box 1 (STOX1), a transcription factor structurally and functionally related to the forkhead family of transcription factors, has been shown to be implicated in the high prevalence of human gestational diseases.^13^, ^14^, ^15^ STOX1 plays a fundamental role in cell proliferation and differentiation. A previous study revealed that overexpression of the transcription factor STOX1 could promote the proliferation of the inner ear epithelial cells via the AKT pathway.^16^ HAM‐1, a homologous to STOX1 in nonmammals, has been reported to prevent neurons undergoing apoptosis and regulate the survival and fate of neural precursors cell.^17^ In addition, Doridot et al^18^ posit STOX1 as a genetic switch in the ROS/RNS balance of trophoblastic cell in pre‐eclampsia. Thus, whether STOX1 can regulate cell proliferation and apoptosis of trophoblast cells is an urgent matter to be investigated. However, the role of STOX1 in the regulation of trophoblastic cell involved in RSA and its mechanism is still obscure. Here, we found downregulation of STOX1 inhibited the proliferation and promoted apoptosis of trophoblast cells via the PI3K/AKT signaling pathway in vitro. This study found a probable mechanism of recurrent spontaneous abortion and might provide a new method for preventing and treating RSA in clinic.

## 2 MATERIALS AND METHODS

### 2.1 Cell culture and reagents

HTR‐8/SVneo trophoblast cells were obtained from Shanghai Institute for Life Science and maintained under standard culture conditions with culture medium and fetal bovine serum (FBS) (Gibco, CA) at 37°C with 95% normal air and 5% CO_2_. LY294002 was acquired from Sigma‐Aldrich (St. Louis, MO). Lipofectamine 2000 was purchased from Invitrogen (CA).

### 2.2 MTT assay

Cell proliferation was assessed using the MTT assays. Cells were incubated in 96‐well plates at a suitable amount of cells. After incubation for 24 hours, the cells were treated with different compounds. The culture medium was removed, and the cells were washed and treated with MTT solution for 4 hours. After incubation, the medium was removed and 200 μL dimethyl sulfoxide was added to each well to solubilize the formazan crystals. Absorbance was measured at 560 nm using a microplate reader. Cell proliferation was expressed as the percentage of MTT reduction. All experiments were performed three times and presented as mean ± standard deviation.

### 2.3 Plasmid construction and transfection

The plasmids were constructed by restriction‐enzyme double digestion and ligation. pcDNA‐AKT and STOX1 were based on the pcDNA backbone with an insertion of the coding region for AKT and STOX1. Transfection was performed using Lipofectamin 2000 reagent (Invitrogen, CA).

### 2.4 Lentivirus‐mediated STOX1 knockdown

The lentiviral expression systems were purchased from System Biosciences (SBI, Mountain View, CA). After transfection, the virus media were harvested, and cells were treated for 72 hours with lentivirus‐mediated STOX1‐ short hairpin RNA (shRNA) and scramble shRNA, respectively.

### 2.5 Flow cytometry

For measuring apoptosis, transfected cells were dual stained with an annexin V‐FITC/7‐amino‐actinomycin D (7‐AAD) kit according to the manufacturer's protocol. The stained cells were immediately analyzed by flow cytometry, and data were collected and analyzed as the percentage of apoptotic cells.

### 2.6 Western blot

Cells were lysed in M‐PER Mammalian Protein Extraction Reagent (Thermo Fisher Scientific, MA), and equal amounts of protein were resolved by sodium dodecyl sulfate‐polyacrylamide gel electrophoresis. Subsequently, the gel‐separated proteins were blotted. The following specific primary antibodies were used to measure proteins: Bcl‐2, Bax, Bim, pAKT, p85, AKT, and glyceraldehyde 3‐phosphate dehydrogenase antibodies. These were obtained from Cell Signaling Technology, Inc (Beverly, MA). Proteins were detected using the Pierce ECL Western blot analysis substrate.

### 2.7 Cell migration assay

Cell migration assay was performed by the transwell chamber assay. Cells were digested and seeded in six‐well culture plates with mitomycin C in medium to inhibit cell proliferation. Then, the cells were plated in serum‐free medium containing 10% FBS in the lower chamber, serving as the chemoattractant. After incubation in 5% CO_2_ at 37°C for 24 hours, the cells that did not penetrate the polycarbonate membrane at the bottom of the chamber were removed with a cotton swab. Then the cells that had invaded through the membrane to the lower surface were fixed with 20% methanol for 20 minutes and stained with 1% crystal violet for 10 minutes, imaged and counted with a microscope (Nikon, Japan).

### 2.8 Dual luciferase reporter gene assays

Cells were cultured in 24‐well plates and transfected with 400 ng of either pGL3 firefly luciferase reporter plasmids together with 25 ng renilla luciferase construct (pRL‐SV40), or 300 ng of wild‐type (wt) or mutant 3′‐untranslated region (UTR) of p85 combined with STOX1 shRNA (shSTOX1). Firefly and renilla luciferase activities were measured 48 hours after transfection using the Dual‐Luciferase Reporter Assay Kit (Promega, Madison, WI) according to the protocol provided by the manufacturer.

### 2.9 Statistical analysis

The data were analyzed using Social Sciences (SPSS) 14.0 software (SPSS Inc, Chicago, IL) and expressed as the means ± standard deviations of three independent experiments. The Student *t* test or analysis of variance was performed to compare the difference between groups. Statistical significance was defined as *P* < 0.05. *P* values were annotated in figure legends.

## 3 RESULTS

### 3.1 Effects of STOX1 downregulation on apoptosis in the trophoblast cells

**Figure 1:**
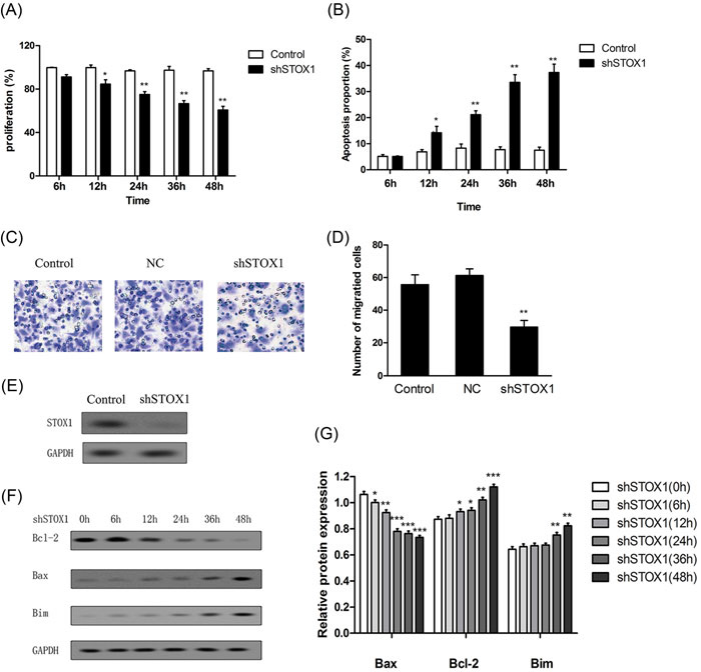
shSTOX1 induced apoptosis in HTR‐8/SVneo trophoblast cells. HTR‐8/SVneo cells were treated with shSTOX1 for 48 hours. A, Cell proliferation was detected using MTT. B, The cells were stained with annexin V‐FITC/7‐AAD, and cell apoptosis was analyzed by flow cytometry. C,D, Cell migration was detected using transwell and the number of migrated cells were analyzed. E, The expression level of STOX1 was examined after treatment with shSTOX1 for 48 hours. F,G, The expressions of Bcl‐2, Bax, and Bim proteins were determined by Western blot analysis, and the relative expression was analyzed versus control. *P < 0.05 versus control, ^**^ *P* < 0.01 versus control, ^***^ *P* < 0.001 versus control. 7‐AAD, 7‐amino‐actinomycin D; shSTOX1, STOX1 shRNA; STOX1, storkhead box 1

STOX1 knockdown has been reported to regulate cell proliferation and apoptosis. To investigate whether STOX1 is involved in the regulation of RSA, STOX1 was downregulated in the trophoblast cells. The downregulation effect of shSTOX1 on the STOX1 expression was examined in HTR‐8/SVneo cells (Figure 1E). The cell proliferation was detected using the MTT assay. The result revealed that cell proliferation was significantly inhibited after the cells were treated with shSTOX1 in a time‐dependent manner, compared with the control. The cells treated with shSTOX1 for 48 hours achieved a lower proliferation and revealed nearly 65% proliferation (Figure 1A). To assess the effect of STOX1 on apoptosis in the trophoblast cells, we used the lentivirus‐mediated shSTOX1 to establish STOX1 knockdown of HTR‐8/SVneo cells. The apoptotic cells of STOX1 knockdown were measured using annexin V‐FITC/7‐AAD. The induction of apoptosis in HTR‐8/SVneo cells was significantly increased in a time‐dependent manner compared with the group (Figure 1B). To study the mechanism of the STOX1 knockdown–influenced apoptosis, the apoptotic proteins were testified. As shown in Figure 1F, STOX1 knockdown resulted in the activation of apoptotic proteins, including Bax and Bim, while reduced the Bcl‐2 protein level in the HTR‐8/SVneo cells. The relative protein expression level of Bcl‐2 was remarkably decreased after treatment with shSTOX1 for more than 6 hours. The relative protein expression levels of Bax and Bim were significantly increased after treatment with shSTOX1 beyond 12 hours and 36 hours, respectively (Figure 1G). Moreover, we assessed the migration of HTR‐8/SVneo cells after treatment with shSTOX1 for 24 hours using transwell assay (Figure 1C and 1D). The result showed that STOX1 knockdown led to a decrease in the migration of HTR‐8/SVneo cells compared with control.

### 3.2 STOX1 knockdown attenuated the PI3K/Akt pathway in the trophoblast cells

**Figure 2:**
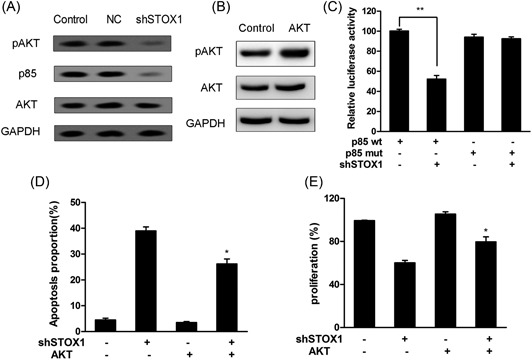
shSTOX1 regulated the PI3K/AKT signaling pathway in HTR‐8/SVneo cells. Cells were treated with shSTOX1 for 48 hours. A, The expressions of pAKT, p85, and AKT proteins were determined by Western blot analysis. B, Cells were treated with shSTOX1 after transfection with pcDNA‐AKT. The overexpression of pAKT was detected in shSTOX1 treated cells. C, Effects of shSTOX1 on 3′‐UTR luciferase reporters of p85 in HTR‐8/SVneo cells. Luciferase activities were calculated normalized to the p85 wt group, ^**^ *P* < 0.01. D, The cells were stained with annexin V‐FITC/7‐AAD and cell apoptosis was analyzed by flow cytometry. *P < 0.05 versus shSTOX1‐independent treatment. E, Cell proliferation was detected using MTT. *P < 0.05 versus shSTOX1‐independent treatment. 7‐AAD, 7‐amino‐actinomycin D; shSTOX1, STOX1 shRNA; UTR, untranslated region; wt, wild‐type

The PI3K/Akt pathway was an intracellular signaling pathway, which played a critical role in cell cycle, proliferation, and apoptosis. To investigate whether STOX1 knockdown affected the PI3K/Akt pathway, shSTOX1 was used to establish STOX1 knockdown in HTR‐8/SVneo cells. As shown in Figure 2A, the pAKT and p85 protein expression levels were significantly decreased after treatment with shSTOX1 for 24 hours compared with the control group. To verify whether shSTOX1 was capable of directly regulating p85, we constructed the wt and mutant 3′‐UTR of p85 in the luciferase vector. The luciferase reporter vectors together with shSTOX1 were transfected into the trophoblast cells. For the wt with p85 reporter, shSTOX1 transfection significantly reduced its relative luciferase activity compared with the group transfected with control shRNA, whereas this effect was abolished in the case of the mutant reporter (Figure 2C). To detect the effect of the PI3K/Akt pathway on the apoptosis induced by the STOX1 knockdown, we constructed the pcDNA‐AKT plasmid and transfected in the HTR‐8/SVneo cells. The expression of pAKT was examined after transfection with pcDNA‐AKT (Figure 2B). The result of cell proliferation revealed that the cell proliferation was remarkably increased after the cells treated with shSTOX1 followed by transfection of pcDNA‐AKT plasmid for 24 hours when compared with treatment with shSTOX1 alone (Figure 2E). Furthermore, the apoptotic cells of STOX1 knockdown were significantly decreased after the cells treated with shSTOX1 followed by transfection of pcDNA‐AKT plasmid for 24 hours when compared with treatment with shSTOX1 alone (Figure 2D).

### 3.3 Overexpression of STOX1 activated the PI3K/Akt pathway in the trophoblast cells

**Figure 3:**
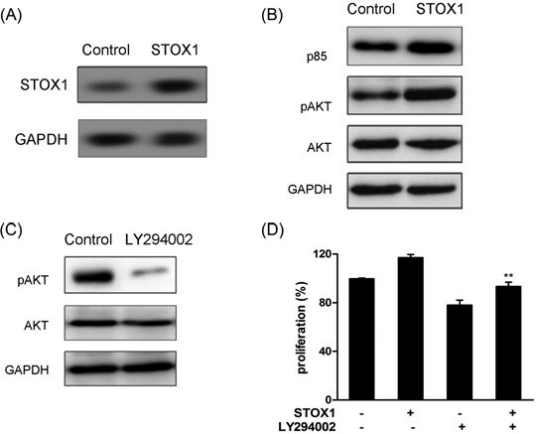
Overexpression of STOX1 regulated the PI3K/AKT signaling pathway in HTR‐8/SVneo cells. Cells were transfected with the STOX1 plasmid for 48 hours. A, The expression level of STOX1 was examined. B, The expressions of pAKT, p85, and AKT proteins were determined by Western blot analysis. C, The expressions of pAKT and AKT proteins were determined after the cells were treated with LY294003. D, Cells were treated with the STOX1 plasmid after pretreatment with LY294002. Cell proliferation was detected using MTT. **P < 0.01 versus shSTOX1‐independent treatment. STOX1, storkhead box 1

To investigate whether STOX1 regulates the PI3K/Akt pathway, STOX1 was upregulated in HTR‐8/SVneo cells. As shown in Figure 3A, the expression level of STOX1 was obviously increased by STOX1 plasmid transfection. Interestingly, the pAKT and p85 protein expression levels were enhanced after transfection with the STOX1 plasmid (Figure 3B). To further verify the effect of STOX1 on PI3K/Akt signaling, the inhibitor of PI3K/Akt signaling LY294002 was used to treat the HTR‐8/SVneo cells. The decreased expression of pAKT was examined after treatment with LY294002 (Figure 3C). Moreover, the result of cell proliferation revealed that cell proliferation was remarkably decreased after the cells treated with STOX1 plasmid followed pretreatment with LY294002 when compared with STOX1 plasmid alone treatment (Figure 3D). These results suggested that the overexpression of STOX1 regulated the PI3K/Akt pathway in the trophoblast cells.

### 3.4 STOX1 knockdown inhibited cell migration and enhanced cell apoptosis through inhibiting the PI3K/Akt pathway in the trophoblast cells

**Figure 4:**
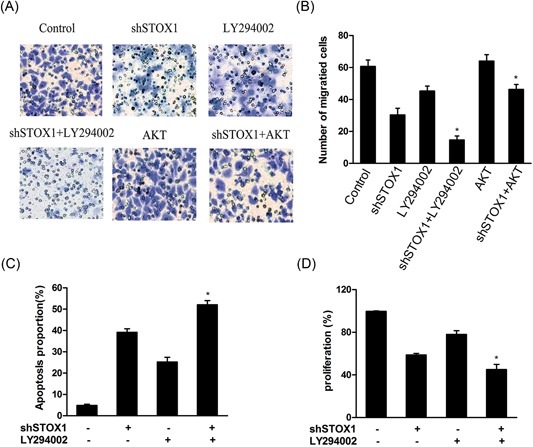
shSTOX1 inhibited cell proliferation and migration through the PI3K/AKT signaling pathway in HTR‐8/SVneo cells. Cells were pretreated with PI3K/AKT inhibitor or pcDNA‐AKT followed by transfection of STOX1 shRNA for 48 hours. A,B, Cell Migration was detected using transwell and the number of migrated cells were analyzed. C, The cells were stained with annexin V‐FITC/7‐AAD and cell apoptosis was analyzed by flow cytometry. D, Cell proliferation was detected using MTT. *P < 0.05 versus shSTOX1‐independent treatment. 7‐AAD, 7‐amino‐actinomycin D; shRNA, short hairpin RNA; shSTOX1, STOX1 shRNA; STOX1, storkhead box 1

**Figure 5:**
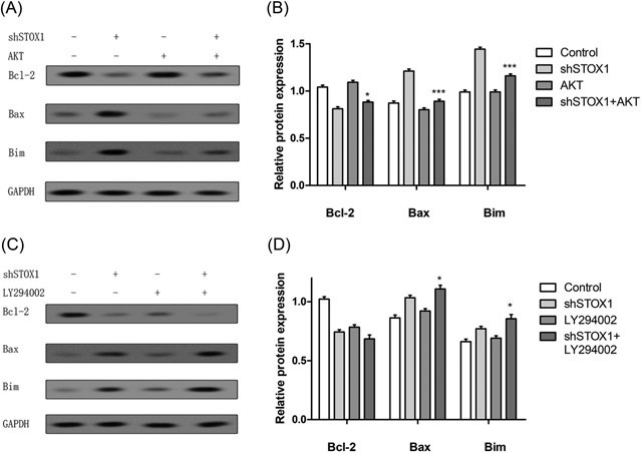
shSTOX1 promoted apoptotic proteins expression in HTR‐8/SVneo cells. Cells were pretreated with PI3K/AKT inhibitor or pcDNA‐AKT followed by transfection of STOX1 shRNA for 48 hours. A‐D, The expressions of Bcl‐2, Bax, and Bim proteins were determined by Western blot analysis and the relative expression was analyzed versus shSTOX1‐independent treatment. *P < 0.05 versus shSTOX1‐independent treatment. ^**^ *P* < 0.01 versus shSTOX1 independently treatment. shSTOX1, STOX1 shRNA; STOX1, storkhead box 1

To study the molecular mechanism of STOX1 on the cell migration and apoptosis, the inhibitors of AKT, LY294002 and pcDNA‐AKT were used to inhibit or activate the PI3K/Akt pathway in the trophoblast cells. As shown in Figure 4A, LY294002 followed STOX1 knockdown treatment significantly decreases the number of migrated cells compared with the shSTOX1‐independent treatment in the trophoblast cells. Whereas pcDNA‐AKT combined with STOX1 knockdown treatment remarkably rescued the decreased migration of STOX1 knockdown in the trophoblast cells (Figure 4B). The result of flow cytometry assay showed that LY294002 could promote the effect of STOX1 knockdown on apoptosis in the trophoblast cells (Figure 4C). Moreover, pcDNA‐AKT combined with shSTOX1 treatment slightly increased the relative protein expression level of Bcl‐2, while significantly decreased the relative protein expression levels of Bax and Bim when compared with shSTOX1‐independent treatment in the trophoblast cells (Figure 5A and 5B). On the other hand, LY294002 combined with shSTOX1 treatment that led to a slight decrease in the relative protein expression level of Bcl‐2, while increased the relative protein expression levels of Bax and Bim when compared with shSTOX1‐independent treatment in the trophoblast cells (Figure 5C and 5D). To further determine the molecular mechanism of STOX1 knockdown on the proliferation, cells were treated with LY294002 to inhibit the PI3K/Akt pathway. The result showed that LY294002 followed by shSTOX1 treatment slightly decreased the cell proliferation compared with shSTOX1‐independent treatment in the trophoblast cells (Figure 4D).

## 4 DISCUSSION

STOX1 is a transcription factor belonging to the forkhead family that is involved in diverse human gestational diseases.^19^, ^20^ A previous study showed that the overexpression of STOX1 could induce transcriptome alteration and pre‐eclampsia in vitro and in vivo.^21^, ^22^ Moreover, a recent study revealed that STOX1 knockdown could inhibit cell proliferation and sphere formation in purified utricular epithelial cells.^18^ However, the effect and molecular mechanism of STOX1 on the RSA are still obscure. Here, this study first investigated the effect of STOX1 on the RSA in vitro. The result revealed that STOX1 knockdown could inhibit the proliferation of the cell in a time‐dependent manner, which was in accordance with the previous results. Furthermore, to estimate whether STOX1 knockdown could affect the apoptosis in the trophoblast cells, HTR‐8/SVneo cells were treated with shSTOX1 for different time periods. The apoptotic cells and related proteins levels of Bax, Bim were significantly increased after the STOX1 knockdown in HTR‐8/SVneo cells. Trophoblast proliferation and migration are essential for establishing and persisting the success of pregnancy. A previous study indicated that dysregulation of indoleamine 2,3‐dioxygenase (IDO) might cause unexplained RSA through inhibiting the trophoblast cell proliferation and migration.^23^ Our results found that STOX1 knockdown could decrease the migration ability of HTR‐8/SVneo cells, which suggested STOX1 downregulation might be associated with RSA.

The PI3K/Akt signaling pathway was reported to play an essential role in cell migration, proliferation, and apoptosis.^24^, ^25^ Our result demonstrated that trophoblast cells expressed lower levels of pAKT and p85 subunits after treatment with shSTOX1 when compared with control. However, overexpression of STOX1 obviously increased the pAKT and p85 protein expression. Moreover, upregulation of the PI3K/Akt pathway by transfection of pcDNA‐AKT plasmid could rescue the cell migration, proliferation, and apoptosis of STOX1 knockdown. However, downregulation of the PI3K/Akt pathway by LY294002 inhibitor significantly suppressed the cell migration and proliferation, while enhanced the cell apoptosis, which was consistent with previous studies.^26^, ^27^, ^28^ In addition, pcDNA‐AKT combined with shSTOX1 treatment slightly increased the relative protein expression level of Bcl‐2, while significantly decreased the relative protein expression levels of Bax and Bim when compared with the shSTOX1‐independent treatment. On the other hand, LY294002 combined with shSTOX1 treatment could lead to a slight decrease in the relative protein expression level of Bcl‐2, while increase the relative protein expression levels of Bax and Bim when compared with shSTOX1‐independent treatment. These observations further hinted that STOX1 knockdown could inhibit cell migration and proliferation, while enhance cell apoptosis through inhibiting the PI3K/Akt pathway in the trophoblast cells.

In conclusion, HTR‐8/SVneo cells showed the decreased proportion of proliferation and migration, while induction of apoptosis after treatment with shSTOX1, which supported the function of STOX1 in human gestational disease. Moreover, specific upregulation of the PI3K/Akt pathway by transfection of pcDNA‐AKT plasmid led to increased cell proliferation, migration, and inhibition of apoptosis in HTR‐8/SVneo cells. Furthermore, inhibition of the PI3K/Akt pathway by specific inhibitors could promote cell apoptosis and aggravatedly suppress cell proliferation and migration of HTR‐8/SVneo cells. In addition, upregulation of the PI3K/Akt pathway could increase the relative expression level of Bcl‐2 and decrease the relative expression levels of Bax and Bim, while inhibition of the PI3K/Akt pathway led to a decrease in the relative expression level of Bcl‐2 and increased expression levels of Bax and Bim. These findings might provide a fundamental mechanism of STOX1 in regulating RSA and could be used to prevent and treat RSA in clinic.

## CONFLICTS OF INTEREST

The authors declare that they have no conflicts of interest.
